# Dancers with non-specific low back pain have less lumbar movement smoothness than healthy dancers

**DOI:** 10.1186/s12938-023-01101-2

**Published:** 2023-04-26

**Authors:** Chai-Wei Lin, Yi-Ting Fang, Jeng-Feng Yang, Bih-Jen Hsue, Cheng-Feng Lin

**Affiliations:** 1 Institute of Allied Health Sciences, College of Medicine, National Cheng Kung University, No. 1, University Rd, Tainan, 70101 Taiwan; 2 Department of Physical Therapy, College of Medicine, National Cheng Kung University, No. 1, University Rd, Tainan, 70101 Taiwan; 3 Physical Therapy Center, National Cheng Kung University Hospital, Tainan, 70101 Taiwan

**Keywords:** Movement, Entropy, Low back pain, Ballet, Dancer

## Abstract

**Background:**

Ballet is a highly technical and physically demanding dance form involving extensive end-range lumbar movements and emphasizing movement smoothness and gracefulness. A high prevalence of non-specific low back pain (LBP) is found in ballet dancers, which may lead to poor controlled movement and possible pain occurrence and reoccurrence. The power spectral entropy of time-series acceleration is a useful indicator of random uncertainty information, and a lower value indicates a greater smoothness or regularity. The current study thus applied a power spectral entropy method to analyze the movement smoothness in lumbar flexion and extension in healthy dancers and dancers with LBP, respectively.

**Method:**

A total of 40 female ballet dancers (23 in the LBP group and 17 in the control group) were recruited in the study. Repetitive end-range lumbar flexion and extension tasks were performed and the kinematic data were collected using a motion capture system. The power spectral entropy of the time-series acceleration of the lumbar movements was calculated in the anterior–posterior (AP), medial–lateral (ML), vertical (VT), and three-directional (3D) vectors. The entropy data were then used to conduct receiver operating characteristic curve analyses to evaluate the overall distinguishing performance and thus cutoff value, sensitivity, specificity, and area under the curve (AUC) were calculated.

**Results:**

The power spectral entropy was significantly higher in the LBP group than the control group in the 3D vector in both lumbar flexion and lumber extension (flexion: *p* = 0.005; extension: *p* < 0.001). In lumbar extension, the AUC in the 3D vector was 0.807. In other words, the entropy provides an 80.7% probability of distinguishing between the two groups (i.e., LBP and control) correctly. The optimal cutoff entropy value was 0.5806 and yielded a sensitivity of 75% and specificity of 73.3%. In lumbar flexion, the AUC in the 3D vector was 0.777, and hence the entropy provided a probability of 77.7% of distinguishing between the two groups correctly. The optimal cutoff value was 0.5649 and yielded a sensitivity of 90% and a specificity of 73.3%.

**Conclusions:**

The LBP group showed significantly lower lumbar movement smoothness than the control group. The lumbar movement smoothness in the 3D vector had a high AUC and thus provided a high differentiating capacity between the two groups. It may therefore be potentially applied in clinical contexts to screen dancers with a high risk of LBP.

## Background

Ballet dance is one of the most popular dance forms in the world [1]. It is a highly technical and physical-demanding dance, which involves extensive end-range lumbar movements in the sagittal plane, such as *arabesque* and *developpé*, and emphasizes fluid, smooth and graceful movements, which require good coordination, strength, and flexibility [2].

Due to the highly technical and physical nature of ballet dance, ballet dancers frequently suffer musculoskeletal injuries, with non-specific low back pain (LBP) being one of the most common [3]. It has been estimated that around two out of every three ballet dancers suffer from non-specific LBP at some point in their dancing careers. This greatly impacts their training and performance and may require medical treatment and/or medication [3]. The effects of LBP on the range and quality of ballet dancer movement have thus received extensive attention in recent decades. The results have revealed the existence of many adaptations in response to LBP, including within- or between-muscle activity pattern change, kinematic and mechanical change, and brain organization change, where all of these adaptations can lead to poor controlled movement [4–6]. Although these adaptations may have an immediate benefit in alleviation pain, they cannot reverse the pain entirely and may even be harmful in the long term [5, 7]. For example, the poor controlled movement caused by these adaptations can lead to pain occurrence or reoccurrence due to a loss of normal spine mobility, increased spinal compression, and pain receptor sensitization and may thus eventually form a vicious cycle [8].

Movement smoothness is a movement quality related to continuality or non-intermittency rather than amplitude and duration [9] and is one of the main characteristics of healthy and trained motor behavior [10]. Many indices, such as jerk-based parameters, have been proposed for evaluating movement smoothness. However, these parameters are not readily adaptable due to practical limitations, such as poor sensitivity, inconvenience, and speed dependence [9, 11, 12]. The power spectral entropy of time-series acceleration, extended from the concept of Shannon entropy, is a useful indicator of uncertainty in random information [13]. In the context of physical movements, a lower power spectral entropy indicates a greater smoothness or regularity. Recent studies have analyzed the power spectra of biomedical signals and have confirmed that the spectrum entropy provides a reliable indicator of movement smoothness in human kinematics [11, 12]. However, the literature lacks information on lumbar movement smoothness in LBP patients compared with asymptomatic participants, despite its clinical importance in ballet moves and many other sporting and artistic performances.

The receiver operating characteristic curve (ROC curve), a mapping of the sensitivity on the y-axis against the “1-specificity” on the x-axis, is widely used in the medical field. For example, the characteristics of the ROC curve, such as the cutoff value and area under the curve (AUC), are commonly used in diagnostic tests to classify subjects into two groups (i.e., with or without disease) [14, 15]. Thus, ROC analyses may also provide a feasible approach for screening high-risk dancers and investigating the underlying factors responsible for the difference between dancers with and without LBP, respectively. However, the literature currently contains very few studies which attempt to differentiate between ballet dancers with and without non-specific LBP using ROC curve analysis techniques [4, 5].

Accordingly, the goals of the present study are as follows: (1) to compare the lumbar movement smoothness in end-range lumbar flexion and extension in female ballet dancers with and without non-specific LBP, respectively, using the power spectral entropy method, and (2) to identify the capacity of ROC curve analyses to evaluate (and differentiate between) the lumbar movement smoothness of female ballet dancers with and without non-specific LBP.

## Results

Forty female ballet dancers participated in the study, of which 23 ballet dancers were with LBP and 17 ballet dancers were without. Five of the participants were identified as extreme outliers (3 in the LBP group and 2 in the control group), and thus their data were excluded from the analysis. No significant difference was found in the age, body height, body mass index, and ballet experience years of the two groups (Table 1). However, a significant difference existed in the body weight.

**Table 1:** Demographic data

	LBP (*n* = 20)	Control (*n* = 15)			
	Mean ± SD	Mean ± SD	*t* value	DOF	*p*-value
Age (years)	24.10 ± 2.20	25.87 ± 4.17	− 1.49	19.80	0.15
Body height (cm)	161.85 ± 3.00	160.27 ± 4.17	1.25	24.28	0.22
Body weight (kg)	53.59 ± 4.76	49.89 ± 3.83	2.46	33.00	0.02*
Body mass index (kg/m^2^)	20.44 ± 1.48	19.44 ± 1.55	1.93	33.00	0.06
Experience (years)	15.35 ± 3.98	16.53 ± 3.31	− 0.96	32.58	0.34
Pain duration (years)	4.53 ± 2.82				

*p < 0.05, significant difference between the LBP and the Control

*DOF* degree of freedom

**Fig. 1:**
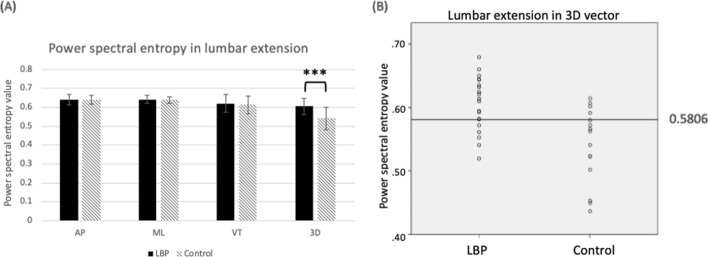
**A** Power spectral entropy in lumbar extension (the error bars represent one standard deviation); **B** Dot plot of 3D vector in lumbar extension with optimal cutoff value of 0.5806 marked by a horizontal line (each dot represents each individual entropy value). ***p < 0.001; *AP* anterior–posterior vector, *ML* medial–lateral vector, *VT* vertical vector, 3D root mean square of AP, ML, and VT vectors

**Fig. 2:**
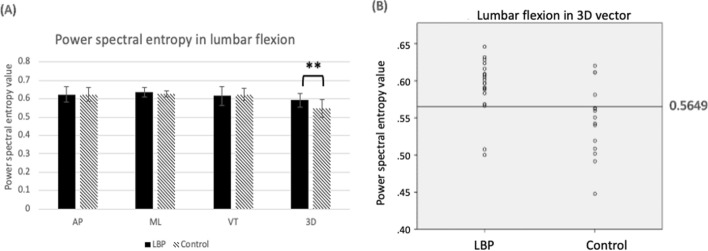
**A** Power spectral entropy in lumbar flexion (the error bars represent standard deviation); **B** Dot plot of 3D vector in lumbar flexion with optimal cut-off value of 0.5649 marked by a horizontal line (each dot represents each individual entropy value). **p < 0.01, *AP* anterior–posterior vector, *ML* medial–lateral vector, *VT* vertical vector, 3D root mean square of AP, ML, and VT vectors

The movement times during end-range lumbar flexion and extension were not significantly different between the two groups (Extension: LBP: 8.01 ± 3.52 s, control: 7.27 ± 2.50 s, *p* = 0.542; Flexion: LBP: 7.25 ± 3.58 s, control: 6.16 ± 1.87 s, *p* = 0.564). Similarly, no significant inter-group differences were found for the power spectral entropies of lumbar flexion and extension in the AP (flexion: *p* = 0.961, effect size (ES) = 0.01; extension: *p* = 0.935, ES = 0.03), ML (flexion: *p* = 0.253, ES = 0.41; extension: *p* = 0.633, ES = 0.11), and VT (flexion: *p* = 0.882, ES = 0.18; extension: *p* = 0.542, ES = 0.16) vectors (Figs. 1 and 2). However, for the 3D vector, the power spectral entropy was significantly higher in the LBP group than the control group in both lumbar extension (*p* < 0.001, ES = 1.24; Fig. 1) and lumbar flexion (*p* = 0.005, ES = 1.06; Fig. 2).

In lumbar extension, the AUC values in the AP, ML, VT, and 3D vectors were 0.517, 0.550, 0.563, and 0.807, respectively. In other words, the 3D vector in the lumbar extension had an 80.7% probability of correctly distinguishing between the LBP group and the control group. The optimal cutoff value was 0.5806 and yielded a sensitivity of 75% and specificity of 73.3%. Thus, the probability of correctly identifying the LBP dancers was equal to 75%, while that of correctly identifying the control group dancers was 73.3%.

In lumbar flexion, the AUC values in the AP, ML, VT and 3D vectors were 0.493, 0.587, 0.483, and 0.777, respectively. Thus, the 3D vector in lumbar flexion had a probability of 77.7% of correctly distinguishing between the LBP group and the control group. The optimal cutoff value was 0.5649 and yielded a sensitivity of 90% and specificity of 73.3%. In other words, the cut-off entropy value had a 90% probability of correctly identifying the LBP patients and a 73.3% probability of correctly identifying the control dancers.

## Discussion

Movement smoothness is an essential requirement of ballet dance and is a critical component of ballet performance. Movement smoothness, or the absence therefore, may provide useful clues as to the physiological condition of the dancer. However, thus far, the literature contains only scant information on the use of objective measures of movement smoothness to distinguish between dancers with non-specific lumbar back pain and those without. Consequently, the present study applied the power spectral entropy of lumbar flexion and extension movements as an objective discriminatory measure. The results showed that the power spectral entropy in the 3D vector (root mean square of the AP, ML, and VT vectors) was significantly higher in the LBP group than in the non-LBP group in both lumbar flexion and lumber extension. In other words, the LBP group showed a significantly lower lumbar movement smoothness than the control group.

Previous studies found that dancers with a longer dance training history showed a greater proportion of somatosensory awareness and shifted the sensorimotor dominance from vision to proprioception, particularly during dynamic movements [16]. Furthermore, patients with chronic LBP presented with impaired proprioception, including a lower sensitivity to detect position change and higher repositioning errors [17, 18]. These findings may suggest a possible mechanism to explain the present observation of a higher entropy value in end-range lumbar extension and flexion movement in the 3D vector in ballet dancers with LBP than in the control group dancers with no LBP.

The present findings are also in line with previous studies that investigated the movement variability in patients with and without LBP. Previous studies showed that, compared to patients without LBP, LBP patients showed increased movement variability as a result of excessive spinal or pelvic movements [19–22]. This finding suggests that patients with LBP have an impaired spinal control ability, which may increase tissue strain and spinal loading during end-range lumbar movements [23]. In constructing theoretical models of movement variability, an optimal variability is required to properly reflect human movements [24]. However, if the actual variability is greater than this optimal variability, the model becomes unstable. Furthermore, if the variability is less than this optimal variability, the model becomes rigid and less adaptable. Therefore, our study provided insight into how the variability changes in LBP dancers than healthy control during spine movements.

Previous studies investigating cervical movement smoothness using the power spectral entropy method found that the smoothness was significantly lower in the 3D vector in chronic neck pain patients than in healthy subjects [13]. Although different parts of the spine were investigated (i.e., the cervical region in [13] and the lumbar region in the present study), the underlying mechanisms may be similar in both cases. Non-specific LBP leads to adaptations in the soft tissues and movement pattern, with increased trunk muscle activation, decreased multifidus activation, uncoordinated kinematic patterns, and altered brain organization [4–6], and this may eventually lead to poor controlled movement. In addition, chronic neck pain patients also show altered motor control, including a change of muscle activation (excessive activation of superficial muscles and inhibition of deep muscles) and kinematic change (delayed feedforward reaction) [25].

In the present study, the between-group difference in the entropy value in the 3D vector was significant, while for the AP, ML, and VT vectors, it was not in both lumbar flexion and lumbar extension. Lumbar flexion/extension movements are a simple task and are thus expected to exhibit a low difference between groups, particularly for ballet dancers, who typically have a significantly higher pain threshold and pain tolerance than normal adults, and a lower inter-segmental coordination variability [26, 27]. Moreover, the 3D vector is a multivector (i.e., the resultant vector of the AP, ML, and VT vectors), and this may also explain why the LBP dancers showed a significantly lower smoothness than the non-LBP dancers only in the 3D vector, i.e., not in the single vectors (the AP, ML, and VT vectors). In particular, the between-group differences in the single vectors may be mitigated by the effects of adaptation and thus be insufficiently large to be significant.

ROC curve analysis provides a useful tool for evaluating the accuracy of medical diagnostic tests, statistical models, and predictive models aimed at classifying subjects into two categories [14, 15, 28]. The present study deliberately chose lumbar movement smoothness as a criterion for differentiating between ballet dancers with and without non-specific LBP, respectively, since movement smoothness is known to have a close relationship with LBP [4, 5]. The ROC curve analysis results showed that the AUC had a high value in the 3D vector (0.807 for extension and 0.777 for flexion), but a low value of 0.5 in the AP, ML, and VT vectors in both lumbar flexion and extension. These findings suggest that lumbar movement smoothness in the 3D vector provides a potential means of discriminating between ballet dancers with and without non-specific LBP (AUC ≥ 0.7). A previous study applied ROC curve analysis to the distance of horizontal single-leg hops in order to identify female collegiate dancers at risk of lower extremity injuries [29]. LBP changes the kinematics and temporal interaction of the lumbar spine during flexion and extension [30] and may therefore result in lower smoothness in LBP dancers. Therefore, simple lumbar flexion and extension movements can be considered as a potential screening movement for dancers with and without LBP, respectively.

Dot plots were used to show the distribution of the entropy value of each group in the two lumbar movements (Figs. 1B and 2B). The majority of the dancers in the LBP group showed a higher entropy value than the optimal cut-off value (0.5806 in lumbar extension and 0.5649 in lumbar flexion). By contrast, almost all of the control groups showed a lower entropy than the optimal cut-off point in both tasks. This may explain why the LBP group showed a high AUC value and why the lumbar movement smoothness in the 3D vector provides a good differentiating capacity between the two groups.

A significant difference was observed between the mean body weights of the two groups but not in body mass index (Table 1). The higher body weight was identified as one of the risk factors for chronic LBP in the general population and workers in a systematic review [31]. However, a study evaluating trunk muscle strength in dance students with and without LBP presented that body weight and body mass index in the LBP group were smaller than those in the no LBP group [32]. Also, a pilot study reported that body mass indexes below 18.5 in female ballet school students could increase lumbosacral pain [33]. Thus, the potential influence of body weight on entropy is still controversial and needs further evaluation. Furthermore, the lumbar movement smoothness was evaluated using easy end-range lumbar flexion–extension movements rather than more complex ballet movements. The dancers with LBP were not having pain during the easy lumbar flexion–extension movements. It is thus possible that the present outcomes may not hold for more realistic ballet dance movements, which have multiple confounding factors, such as skill level, muscle strength, range of motion, previous injury history of the lower extremities and pelvis region, and so on. Moreover, different directions of trunk movement such as trunk lateral flexion and rotation should be incorporated into the future study.

## Conclusions

The power spectral entropy of the time-series acceleration provides a suitable and sensitive indicator for quantifying the movement smoothness in ballet dancers with and without non-specific low back pain. The lumbar movement smoothness is significantly lower in non-specific low back pain dancers than in control group dancers in both end-range lumbar flexion and end-range lumbar extension.

Overall, the present results suggest that simple movements such as full lumbar flexion and extension performed in conjunction with portable devices such as inertial measurement unit systems may provide a feasible and more objective approach for screening dancers with a high risk of LBP in clinical settings than traditional subjective observations. However, further investigation is required to clarify the underlying causes responsible for the between-group differences observed in the present study in order to better prevent the possible occurrence or reoccurrence of LBP.

### Methods

Female ballet dancers were recruited through a flyer. The inclusion criteria were specified as an age of more than 20 years old and a classical ballet training background of at least 10 years. For the LBP group, the dancers were assigned one of three different LBP diagnoses based on an interview process, namely: (1) pain region between the lower margin of ribs and buttock fold and no specified pathology (for example, herniated intervertebral disk or spondylolisthesis); (2) with an episode duration of more than three months and consistent or intermittent pain after first episode; and (3) average pain level greater than or equal to 3 out of 10 points on a numerical pain rating. For the healthy control group, the participants had no relevant history of back pain or any other form of discomfort that might have affected their dancing movement over the past 2 years.

All of the participants with severe lower extremity injuries in the past year that affected their daily lives were excluded from the study. Those who were pregnant, or had a history of cardiovascular disease, a body mass index of more than 30, or neurological signs or red flags for lumbar disorders (e.g., numbness and pain referred to lower extremities, malignancy, infection, systemic steroid use, and so on) were similarly excluded from the research.

Prior to data collection, initial screening was performed through an interview process. The eligible participants attended a baseline evaluation and were introduced to the experimental procedure. They were then requested to sign a consent form approved by the Institutional Review Board of National Cheng Kung University Hospital (IRB number: A-BR-108-113-T). Finally, each participant completed a questionnaire on their basic demographic data and dance experience/history.

Each participant performed three repetitions of trunk full extension followed by full flexion starting with standing in the full lumbar flexion posture. The knees were kept straight with no restriction over pelvis motion. A motion analysis system consisting of eight infrared cameras (Kestrel 4200 Cameras, Cortex System, Motion Analysis Corporation, USA) was used to record the three-dimensional trajectories of two reflective markers attached on the T12 and S1 spinous processes to record the whole lumbar spine movement. Sampling was performed at a rate of 100 frames per second.

The kinematics of the lumbar movement were analyzed in the second repetition of each trial in order to minimize the effects of movement errors in the starting and ending movements, respectively. For each participant, the raw time-series speed data of each marker was recorded by the motion analysis system, and the lumbar acceleration was calculated in three vectors, namely anterior–posterior (AP), medial–lateral (ML), and vertical (VT). The lumbar acceleration in the 3-directional (3D) vector (the root mean square of the AP, ML, and VT vectors) was also evaluated. To calculate the spectral entropy of the time-series acceleration data, the lumbar acceleration in each vector was first smoothed by a Butterworth 4th order low pass filter with a 10 Hz cut-off frequency using a Hanning window function to avoid spectral leakage. The smoothed lumbar acceleration data were then transformed into frequency-domain acceleration data via fast Fourier transformation. The power spectral density of the lumbar acceleration in the frequency domain was calculated and normalized to the interval of [0,1]. Finally, the power spectral entropy of the lumbar time-series acceleration in each vector was computed by the following formula:

$${\text{Lumbar}}_{acc} = \frac{{{\text{d}}y}}{{{\text{d}}x}}\left( {\frac{{{\text{d}}y}}{{{\text{d}}x}}\left( {T12_{{{\text{position}}}} - S1_{{{\text{position}}}} } \right)} \right)$$

All the above processes were calculated using a self-developed program written in MATLAB version R2020b (The MathWorks, Natick, MA, USA). The group means and standard deviations (SD) were calculated for each demographic and dependent variable. Extreme outliers based on the boxplot of entropy score were excluded from the analysis if the values were below the 1st quartile—1.5*(interquartile range) or above the 3rd quartile + 1.5*(interquartile range). After verifying the data normality assumption by means of the Shapiro–Wilk test (*p* < 0.05), a parametric independent sample *t* test was employed to test the differences between the two groups. The spectral entropies of the lumbar extension in the ML and VT vectors, and lumbar flexion in the AP, VT, and 3D vectors, were not normally distributed. The movement times during end-range lumbar flexion and extension were similarly not normally distributed in either group. Thus, a Mann–Whitney *U* test was used to detect the differences between groups. The significance level was set as *p* < 0.05 and the effect size was calculated with the value of Cohen’s d. Finally, ROC analyses were performed to determine the optimal cutoff values and AUCs for the power spectral entropies in the lumbar extension and lumbar flexion tasks. All of the statistical analyses were performed using SPSS version 17.0 (SPSS Inc., USA).

## Data Availability

The data sets used and/or analyzed for the current study are available from the corresponding author on reasonable request.

## Abbreviations

3D: Three-directional
AP: Anterior–posterior
AUC: Area under the curve
LBP: Low back pain
ML: Medial–lateral
ROC: Receiver operating characteristic
VT: Vertical
